# Use of Skin Cancer Procedures, Medicare Reimbursement, and Overall Expenditures, 2012-2017

**DOI:** 10.1001/jamanetworkopen.2020.25139

**Published:** 2020-11-10

**Authors:** Pranav Puri, Sujith Baliga, Mark R. Pittelkow, Puneet K. Bhullar, Aaron R. Mangold

**Affiliations:** 1 Mayo Clinic Alix School of Medicine, Scottsdale, Arizona; 2 Department of Radiation Oncology, The Ohio State University, Columbus; 3 Department of Dermatology, Mayo Clinic, Scottsdale, Arizona

## Abstract

This cohort study describes recent trends in use and payment rates and in overall expenditure for skin cancer procedures in the Medicare Part B population in the United States.

## Introduction

Skin cancers represent the most common malignant neoplasms in the United States and account for more than $8 billion of health expenditure annually.^1^ Because the US population is aging, the incidence of skin cancers is increasing.^2^ In addition to topical chemotherapy, procedural treatments for skin cancers include Mohs micrographic surgery (MMS), simple surgical excision, and shave excision as well as destructive modalities including laser surgery, electrosurgery, and cryosurgery. Medicare payments vary widely across these types of procedures.^3^ However, little is known about how variations in payment correspond to the use of different skin cancer procedures. This study describes recent trends in payment rates, use rates, and overall expenditure for skin cancer procedures in the Medicare Part B population.

## Methods

For this cohort study, we grouped procedures into the following categories using Healthcare Common Procedure Coding System codes: simple excision, MMS, shave excision, and destruction of malignant lesions. Using the Medicare Physician Supplier and Other Provider Public Use File,^3^ we aggregated the volume of services, number of clinicians (physicians, physician assistants, and nurse practitioners), and mean Medicare Part B payments from January 1, 2012, to December 31, 2017. We adjusted for inflation by converting payments to 2017 dollars. We calculated the mean number of providers for each Healthcare Common Procedure Coding System code within the procedure category. We defined use rates as the total number of services in the procedure category divided by the year’s Medicare Part B population. This study was exempt from Mayo Clinic institutional review board approval because the data were publicly available and deidentified. This study followed the Strengthening the Reporting of Observational Studies in Epidemiology (STROBE) reporting guideline for cohort studies. We used JMP, version 14 (SAS Institute Inc) for data analysis.

## Results

From 2012 to 2017, MMS services had the highest mean payment ($378.71; range, $41.24-$466.93) and shave excisions had the lowest ($70.99; range, $15.58-$135.24) (Table). During this period, payment rates declined for each procedure class with the exception of shave excision. The use rates of simple excision, shave excision, and destruction of malignant lesions all declined from 2012 to 2017. However, during this period, the use rate of MMS increased 21% from 3554 per 100 000 Medicare beneficiaries to 4293 per 100 000 Medicare beneficiaries (Figure). From 2012 to 2017, total expenditures for simple excision and destruction of malignant lesions declined, whereas total expenditures for MMS and shave excision increased. Total Medicare spending on skin cancer procedures increased 9% from $743 222 614 in 2012 to $806 392 161 in 2017. This was primarily associated with an increase of $83 363 703 (18%) in expenditures for MMS. Expenditures for MMS represented 61% of overall spending on skin cancer procedures in 2012, and this proportion increased to 67% in 2017.

**Table.** Skin Cancer Procedure Use, Payment, and Total Expenditure, 2012-2017^a^

Year	Services, No.	Clinicians, No.	Medicare				
			Use rate per 100 000 beneficiaries	Payment, mean (range), $	Total expenditure, $	Overall skin cancer procedure expenditure, $	Share of overall skin cancer surgery expenditure, %
**Simple excision**							
2012	809 216	5121	2457	155.32 (67.91-343.76)	125 686 685	743 222 614	17
2013	806 413	5029	2433	152.73 (67.22-394.76)	123 164 770	765 640 334	16
2014	800 086	4908	2411	147.24 (68.11-387.62)	117 809 702	757 342 759	16
2015	793 094	4754	2383	147.24 (70.60-391.32)	116 779 467	792 069 533	15
2016	801 283	4658	2377	143.98 (65.08-381.03)	115 375 675	824 099 471	14
2017	784 840	4515	2338	137.81 (63.95-369.63)	108 158 113	806 392 161	13
**Mohs micrographic surgery**							
2012	1 170 682	990	3554	387.45 (43.10-466.93)	453 582 606	743 222 614	61
2013	1 227 926	1034	3704	378.17 (41.24-453.20)	464 361 335	765 640 334	61
2014	1 250 876	1051	3769	373.36 (43.47-445.94)	467 033 139	757 342 759	62
2015	1 316 146	1097	3954	380.97 (44.08-452.84)	501 418 473	792 069 533	63
2016	1 415 383	1131	4198	379.41 (44.51-444.90)	537 016 751	824 099 471	65
2017	1 440 886	1168	4293	372.88 (43.42-436.45)	537 272 681	806 392 161	67
**Shave excision**							
2012	922 097	4267	2799	59.01 (15.58-113.64)	54 413 148	743 222 614	7
2013	933 443	4216	2816	75.12 (17.31-135.24)	70 117 437	765 640 334	9
2014	928 202	4153	2797	73.03 (17.53-132.39)	67 785 854	757 342 759	9
2015	911 051	4080	2737	74.63 (17.89-133.53)	67 988 970	792 069 533	9
2016	920 578	4061	2730	73.32 (17.29-127.44)	67 490 623	824 099 471	8
2017	891 386	3986	2656	70.83 (17.10-124.78)	63 137 492	806 392 161	8
**Destruction of malignant lesion**							
2012	881 705	2347	2677	124.24 (42.82-282.70)	109 540 173	743 222 614	15
2013	883 964	2407	2667	122.18 (42.04-280.41)	107 996 792	765 640 334	14
2014	892 372	2482	2689	117.34 (40.84-273.19)	104 714 061	757 342 759	14
2015	896 669	2506	2694	118.08 (41.51-273.24)	105 882 620	792 069 533	13
2016	915 288	2538	2715	113.87 (37.44-272.17)	104 216 421	824 099 471	13
2017	879 437	2586	2620	111.23 (35.31-262.19)	97 823 872	806 392 161	12

^a^ All payment values are inflation adjusted to 2017 dollars.

**Figure.**
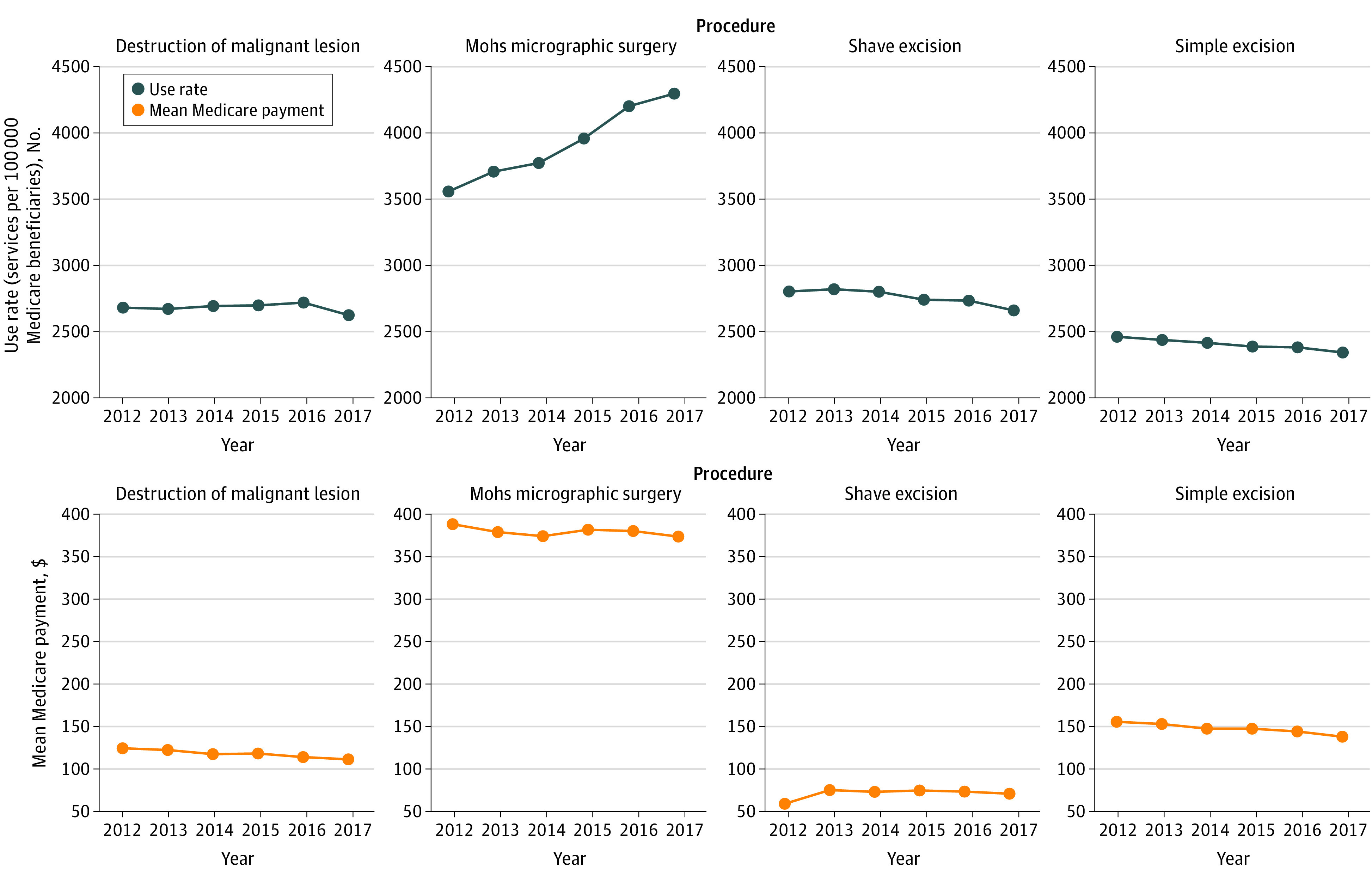
Use Rates and Payment Rates by Skin Cancer Procedure, 2012-2017

## Discussion

The results of this cohort study suggest that increased use of MMS has displaced use of other skin cancer procedures in the Medicare population. Medicare payment rates for MMS are more than double those for simple excisions. Thus, from an economic perspective, Medicare fee-for-service pricing incentivizes use of MMS compared with other modalities.

In 2012, the American College of Mohs Surgery developed appropriate use criteria at least in part to reduce overuse.^4^ However, despite the adoption of appropriate use criteria, our study shows that MMS use rates have steadily increased in the Medicare population. This has resulted in inflation-adjusted growth in spending on skin cancer procedures.

This study has several limitations. First, because this study lacks patient-level clinical data, we cannot determine the appropriateness or outcomes of procedures. Second, the trends we describe may not be generalizable outside the Medicare Part B population. Third, this study did not account for ancillary costs such as charges for repair, skin flap creation, and pathological examination.

Given the increasing incidence of skin cancer, further study is needed to evaluate whether the increasing use of MMS is improving value. Policy makers could potentially titrate incentives by linking payments for MMS to adherence with appropriate use criteria. Similarly, Medicare could provide a fixed, bundled payment for treatment of newly diagnosed skin cancers, thereby incentivizing more cost-effective procedures.
